# Comparing pulse oximeter performance using a common functional tester versus controlled desaturation studies on healthy participants

**DOI:** 10.1007/s10877-025-01381-0

**Published:** 2025-11-14

**Authors:** Seif Elmankabadi, Jake Dove, Ella Behnke, Yu Celine Chou, Lily Ortiz, Gregory Leeb, Isabella Auchus, Danni Chen, John Feiner, Tyler J. Law, Philip E. Bickler, Shamsudini Hashi, René Vargas Zamora, Fekir Negussie, Ronald Bisegerwa, Michael Bernstein, Michael S. Lipnick

**Affiliations:** 1 Department of Anesthesia and Perioperative Care, Zuckerberg San Francisco General Hospital and Trauma Center, University of California San Francisco, San Francisco, CA USA; 2 University of California San Francisco Hypoxia Research Laboratory, San Francisco, CA USA; 3 University of California San Francisco School of Medicine, San Francisco, CA USA; 4 University of California San Francisco Center for Health Equity in Surgery and Anesthesia, San Francisco, CA USA; 5 Acute Care and Monitoring, Medtronic, Minneapolis, MN USA; 6 Department of Anesthesia and Critical Care, Makerere University, Kampala, Uganda; 7 Association of Anesthesiologists of Uganda, Kampala, Uganda

**Keywords:** Pulse oximeter, Functional tester, Hypoxemia studies

## Abstract

Functional testers are designed to evaluate select pulse oximeter characteristics but are often misused to validate device accuracy, potentially providing false reassurance. This study evaluated whether the Fluke ProSim8 (FPS8) could accurately predict oximeter performance during human controlled desaturation studies or identify performance differences under low signal conditions. 12 oximeters were tested using two FPS8 protocols: (1) an ‘SpO₂ plateau’ protocol which mimicked controlled desaturation studies by evaluating device performance over a range of simulated SpO_2_ (70–100%), and (2) a ‘signal space’ protocol designed to assess device accuracy under varying modulation and transmission conditions. Each device also underwent controlled desaturation testing in healthy adults. Six of the 12 oximeters passed (ARMS *≤* 3%) the SpO₂ plateau protocol; however, three of these failed (ARMS > 3%) human testing. At lower simulated saturations, most devices overestimated SpO₂. In the signal space protocol, oximeters performed well under high signal conditions, but many failed to produce readings or showed SpO₂ errors > 3% under low signal conditions. On average, oximeters failed to generate a reading 20.2 ± 7.2 times out of 60 attempts. Ten devices passed (ARMS < 3%) the signal space protocol, but two of these failed human testing. Oximeter performance on the FPS8 did not correlate with human performance (R² = 0.08 for the plateau protocol; R² = 0.01 for the signal space protocol). The FPS8 did not reliably predict oximeter accuracy in human desaturation studies or under low signal conditions; current functional tester protocols are limited in predicting real-world oximeter performance.

## Background and objectives

Select functionality of pulse oximeters, including identification of optical, electrical, and software issues can be assessed in vitro by ‘functional testers’ (Table 1) [1–5]; consequently, functional testers are immensely helpful tools for biomedical engineers as well as pulse oximeter developers.

**Table 1:** Commercially available pulse oximeter simulators/functional testers

Manufacturer & model	Manufacturer guidelines	Studies that used specified simulators	Probe compatibility
Fluke Biomedical: ProSim 8	“Provide an optical signal to verify that the electronics within the pulse oximeter probe are functional.” [1] “This Product is not intended to be used to calibrate medical equipment.” [1]	[6–20]	Transmittance (Artificial Finger)
Fluke Biomedical: ProSim SPOT Light SpO_2_ Pulse Oximeter Tester	“To test and verify the basic operation of patient monitoring devices or systems used to monitor SpO_2_.” [2] “To verify the electronics inside the pulse oximeter sensor are functional.” [2]	[21–24]	Transmittance (Artificial Finger)
Fluke Biomedical: Index 2 XLFE Series Pulse Oximeter Tester*	“provides simulations that allow thorough testing of the complete pulse oximeter, including the optical sensors.” [3] Provides “a reliable way to gauge the condition and performance of standard pulse oximeters.” [3] “allow measuring pulse oximeters currently on the market against widely accepted performance standards” [3]	[25]	Transmittance (Artificial Finger)
BioTek Instruments: BioTek Index 1*	“Capable only of testing for shorts, continuity, opens and LED functionality” [26]	[26, 27]	Transmittance (Simulated Finger)
The Electrode Company: Lightman*	“The Lightman is intended to test the optical and electrical properties of a pulse oximeter probe including the wavelength of the light emitting diode by means of a micro spectrometer.” [28]	[28, 29]	Transmittance (Artificial Finger; referred to as the ‘active area’)
WhaleTeq: PPG-2R-880/PPG-2R-940	“suitable for the development and verification of reflectance PPG products such as wearable devices to test performance.” [5]	[5, 30]	Reflectance
Contec Medical Systems: Contec SpO_2_ Simulator MS100 Pulse	MS100 “can perform a series of tests for the oximeter by simulation means, and gives cognizance of veracity about the oximeter.” [31]	[32, 33]	Transmittance (Simulated Finger)

This table describes the manufacturer, model, manufacturer guidelines, and studies that have used the simulators, and probe compatibility (reflectance, transmittance) of commercially available in vitro functional testers. The bolded text highlights the functional tester’s functionality according to the manufacturer manual or source listed. Some of the in vitro functional testers described in the table are no longer commercially available and are denoted with an *.

Pulse oximeters work by shining at least two different wavelengths of light, red and infrared (IR), through a tissue. When blood passes through the arteries with each heartbeat, it changes the amount of light absorbed. At high blood oxygen saturation (SaO_2_) levels, the oxygen-rich hemoglobin in the blood absorbs IR light more than red light, so the red light is modulated (or absorbed) less by the blood. At low oxygen saturations, the deoxygenated hemoglobin absorbs red light more than IR light. Pulse oximeters measure the ratio of red to infrared light absorption during pulsatile blood flow compared to non-pulsatile blood flow, whereby the device can determine the relative proportion of oxygenated hemoglobin to deoxygenated hemoglobin, allowing it to estimate blood oxygen levels (SpO_2_).

For an oximeter to work properly, it must be able to accurately measure how much the detected red and IR light signals change across low to high levels of light absorption and across different blood flow conditions (i.e. poor to strong blood flow). For example, a darkly pigmented, thick finger can reduce the overall strength of the light signal (low transmission), so less light will pass through (i.e. less transmitted light). However, if the finger is well perfused, the changes in light absorption caused by the pulsing blood (percent modulation) could still be strong enough for the pulse oximeter to obtain an accurate reading. Percent modulation represents the changes in light absorption, and percent transmission reflects how much light passes through the tissues. As a result, percent modulation and percent transmission can be used to create a two-dimensional space that captures the majority of signals a pulse oximeter can measure.

Functional testers are useful tools that can challenge oximeters over this two-dimensional signal space by creating synthetic optical signals that mimic the pulsatile nature of blood flow, changes in light absorption, and the amount of light detected, thereby simulating an SpO_2_ value.

Functional testers can vary both percent modulation and transmittance in an attempt to simulate detected probe signals. The parameters of the synthetic optical signals can be altered with the functional tester until the noise floor of the pulse oximeter is reached, which is when the devices begin to consistently either report a flag or not report a reading. Essentially, these testers can evaluate pulse oximeter function under inadequate signal conditions, which can be useful in the development phase of a new device [34]. As a result, manufacturers sometimes use functional testers as an adjunct to human studies to simulate extreme conditions that cannot be reliably or safely recreated in a laboratory or clinical setting.

Functional testers are also useful to biomedical engineers seeking to interrogate gross functionality of oximeters in clinical use (e.g. intact electrical hardware). However, functional testers are often incorrectly used to assess the accuracy of pulse oximeter saturation (SpO_2_), a capability most do not possess, which can lead to inaccurate conclusions regarding device performance [21, 26, 27]. As summarized in Table 1, numerous studies have used these testers to draw conclusions about device accuracy or clinical performance. In the wake of COVID19, multiple aid non-governmental organizations (NGOs) implemented functional testers to determine which pulse oximeters have the ‘best’ accuracy to inform procurement decisions. This is problematic because functional testers cannot reproduce the complex opto-physiological interactions that occur in human tissue, such as variations in skin pigmentation and perfusion. Consequently, their results can be misleading and may contribute to the distribution of poorly validated or inaccurate oximeters. This issue is particularly concerning in low- and middle-income countries, where limited access to regulatory testing and validated devices increases reliance on low-cost oximeters that may have been evaluated solely using in vitro testers.

Moreover, the current International Organization for Standardization’s - ISO 80601-2-61:2017 guidelines state that functional testers cannot be used to assess the SpO_2_ accuracy of pulse oximeters or determine their proper calibration [35]. To obtain regulatory clearance and conformance for clinical use, pulse oximeters must undergo performance validation studies in healthy humans during controlled hypoxemia in a laboratory setting (i.e. controlled desaturation studies) [35–37]. These studies control inspired oxygen levels and expose participants to stable hemoglobin saturation (SaO_2_) levels of 70–100%. During this process, simultaneous SpO_2_ is measured and compared to SaO_2_ values obtained from blood specimens, which are analyzed using a co-oximeter–the clinical gold standard for measuring oxygen saturation. Controlled desaturation studies include numerous components that in vitro testing cannot replicate.

This study characterized and compared the performance of 12 pulse oximeters on a popular functional tester as well as in a controlled laboratory desaturation study on healthy volunteers [36, 38]. The primary objective was to determine whether a commonly used functional tester, the Fluke ProSim8 (FPS8), could accurately predict pulse oximeter performance in healthy adult volunteers during controlled desaturation studies. The secondary objective was to assess if the FPS8 could identify performance differences among oximeters under low signal conditions, such as low modulation and transmission. We hypothesized that oximeter performance on the functional tester would not predict oximeter performance in humans during controlled hypoxemia nor would it identify performance differences under low signal conditions.

## Methods

The FPS8 was selected because it is the most widely used functional tester in published pulse oximeter validation studies, allowing for better alignment with prior methodology and improved comparability across studies (Table 1). We used the FPS8 with the SPOT SpO_2_ accessory (an optical interface) (Fluke Biomedical; Everett, Washington, USA) connected to an open source terminal program (Tera Term) of a PC computer to allow greater customization of modulation and transmitted light settings than possible from the FPS8 built-in control panel. The FPS8 was connected to the PC via USB-to-USB protection isolation board (Teyleten Robot ADUM3160 Module) to isolate and limit the electrical noise. During the test, the FPS8 always ran on a full battery.

Pulse oximeters were selected based on their popularity in global markets as well as a wide range of performance as reported by the OpenOximetry.org database at the time of the study [38]. Eleven fingertip pulse oximeters (price range: $20–299 USD) and one handheld device ($5999 USD) were selected [38].

We implemented two testing protocols using the functional tester: (1) an ‘SpO_2_ plateau’ protocol that tested devices over a range of simulated SpO_2_ 70–100%; and (2) a ‘signal space’ protocol that tested devices at different percent modulation and transmission combinations. For both protocols, the FPS8 settings included a pulse amplitude of 3%, a ‘medium finger size,’ a heart rate of 60 beats per minute, and the default Nellcor R-curve. Both the respiration and ambient light settings of the FPS8 were turned off. The Nonin CO-Pilot, Masimo Mightysat, and Nonin Onyx Vantage 9590 had manufacturer-specific R curves (referred to as “Type” on the FPS8) pre-programmed onto the FPS8. The performance for these three devices was evaluated on the SpO_2_ plateau protocol both with and without their manufacturer-specific R curves.

The SpO_2_ plateau protocol was designed to mimic a range of SpO_2_ similar to that recommended by ISO 80601-2-61 for controlled desaturation studies in healthy human adults [35]. The protocol started with a set SpO_2_ of 100% on the FPS8, then decreased the SpO_2_ by increments of 5% until reaching a final set point of 70% on the FPS8. At each SpO_2_ level, a 30-second stabilization period was allowed before data collection began. During the subsequent 60-second data recording period, the highest and lowest SpO_2_ values reported by each device were recorded. The mean SpO_2_ was calculated based on these highest and lowest values, and the difference between this mean SpO_2_ and the set SpO_2_ on the FPS8 was measured. This SpO_2_ plateau protocol was used on each device tested. The error for each device was assessed at each set point by calculating the difference between the oximeter reported SpO₂ and the set SpO_2_ on the FPS8. The root mean square error (ARMS) for each device was calculated by taking the square root of the mean percentage differences between the mean SpO_2_ on the oximeter and the set SpO_2_ on the functional tester (or the SaO_2_ during human testing), squared.

$$\mathrm{ARMS} = \sqrt {\frac{1}{n}\sum _{{i = 1}}^{n} (SpO_{{2i}} - SaO_{{2i}} )^{2} }$$

The error was calculated to assess the bias between the reported SpO₂ and the set point on the FPS8, while the ARMS was used to evaluate the overall accuracy and precision of the devices, taking into account both the magnitude and variability of errors.

The signal space protocol tested each device under a fixed range of modulation from 10% to 0.02% (decreased by a factor of 2; including 10, 5, 2.5, 1.25, 0.625, 0.313, 0.156, 0.078, 0.039, 0.020) and transmission values from 10% to 0.375% (decreased by a factor of 2; including 10, 5, 2.5, 1.25, 0.75, 0.375) to evaluate a target SpO_2_ of 90%. “High signal space” referred to transmission values *≥* 2.5% and percent modulation values *≥* 0.313. “Low signal space” referred to transmission values < 2.5% and percent modulation values < 0.313. A ‘failure’ for device performance was determined if the pulse oximeter was unable to generate an SpO_2_ reading at a certain modulation and transmission pair. The frequency of the device’s reading failure due to poor simulator signal was also recorded.

These settings and thresholds were selected by pulse oximeter engineers based on observed device performance, including conditions under which the devices commonly performed well and those under which they failed. The parameter range was intentionally designed to include low transmission and modulation values not typically achievable in healthy volunteer studies but are more likely to occur in clinical scenarios where disease states compromise signal quality. When excluding the points that did not generate an SpO_2_, the ARMS was calculated for both the high and low signal space. The ARMS and error for each oximeter was calculated in a similar manner to the SpO₂ plateau protocol, but the signal space protocol had a fixed SpO_2_ of 90% on the FPS8. The absolute value of this error was then assessed to identify devices that consistently performed poorly or deviated significantly from the reference values.

Each oximeter also underwent performance assessment in a controlled laboratory desaturation study on healthy adult volunteers across an SaO_2_ 70–100% to determine ARMS, consistent with 2017 ISO 80601-2-61 and 2013 US Food and Drug Administration (FDA) guidance for pulse oximeter testing [35–38]. These tests were conducted as part of the Open Oximetry project, and we used the latest ARMS values available in the Open Oximetry database at the time of publication [36, 38]. The Open Oximetry database is actively being updated as more data are available, so the sample sizes for each device may differ [38]. At the time of data analysis, each oximeter was evaluated in at least 24 adult participants (age 18–45 years old), with >40% male and >40% female, with at least 25% of participants in each skin color grouping (light, medium or dark) as defined by both Monk Skin Tone Scale and individual typology angle (i.e. the most widely published objective surrogate for melanin content) [39]. Written informed consent was obtained from each adult volunteer prior to their participation in lab desaturation studies, and the study protocol was approved by the University of California San Francisco Institutional Review Board (IRB #23–40212).

For both protocols, “pass” was defined as ARMS *≤* 3%, which is the threshold used for controlled desaturation studies as stipulated by FDA 2013 (ARMS ≤ 3%). Performance “failure” was defined as ARMS > 3%. We conducted a linear regression analysis to compare the ARMS values from the SpO₂ plateau and signal space protocols with the ARMS from the controlled desaturation studies. The coefficient of determination (R²) was calculated for each regression to evaluate the correlation between the ARMS from the FPS8 testing and the ARMS from the controlled desaturation studies.

## Results

### SpO_2_ plateau protocol

Of the 12 devices tested, only six ‘passed’ the in vitro SpO_2_ plateau protocol with an ARMS threshold of *≤* 3% (Table 2).

**Table 2:** Comparison of oximeter performance on a functional tester vs. humans during controlled hypoxemia

Device	Cost	ARMS in healthy subjects (SaO_2_ 70%−100%)	ARMS from FSP8 SpO_2_ plateau protocol	ARMS from FSP8 signal space protocol	Device reading failures from signal space protocol
Nonin CO-Pilot (Nellcor R-curve)	$5999	1.39	3.68	2.2	19
Nonin CO-Pilot (Manufacturer-specific R-curve)	$5999	1.39	3.49	N/A	N/A
Masimo Mightysat Rx Fingertip Pulse Oximeter (Nellcor R-curve)	$299	2.1	1.64	0.8	15
Masimo Mightysat Rx Fingertip Pulse Oximeter (Manufacturer-specific R-curve)	$299	2.1	0.19	N/A	N/A
Nonin Onyx Vantage 9590 (Nellcor R-curve)	$200	2.24	2.61	1.8	14
Nonin Onyx Vantage 9590 (Manufacturer-specific R-curve)	$200	2.24	0.42	N/A	N/A
Walgreens MD300CN350R	$30	2.27	3.91	1.0	23
Zacurate CMS 500DL	$23	2.5	4.21	0.9	14
Zacurate 500 C	$23	2.51	3.91	0.6	24
Walgreens Oxywatch C20	$35	2.6	4.09	0.6	21
ChoiceMMed MD300CN340	$25	2.7	4.21	3.4	36
CONTEC Med CMS-50 M	$20	2.82	2.39	1.0	31
Bodymed BDMOXMTRBLK	$25	3.26	2.62	1.5	17
Roscoe POX-ROS	$21	4.3	2.15	3.6	20
Biolight M70	$60	5.08	0.96	1.2	8

This table shows root mean square error (ARMS) for each oximeter during a controlled desaturation study in humans (SaO2 range 70–100%), during the functional tester SpO2 plateau protocol (SpO2 range 70–100%), and during the functional tester signal space protocol. Devices with manufacturer-specific R-curves underwent testing with their R-curve as well as the default Nellcor R-curve during the SpO2 plateau protocol. The frequency of the device’s reading failure was calculated by the number of occurrences at which the pulse oximeter device was unable to produce a SpO2 reading.

**Fig. 1:**
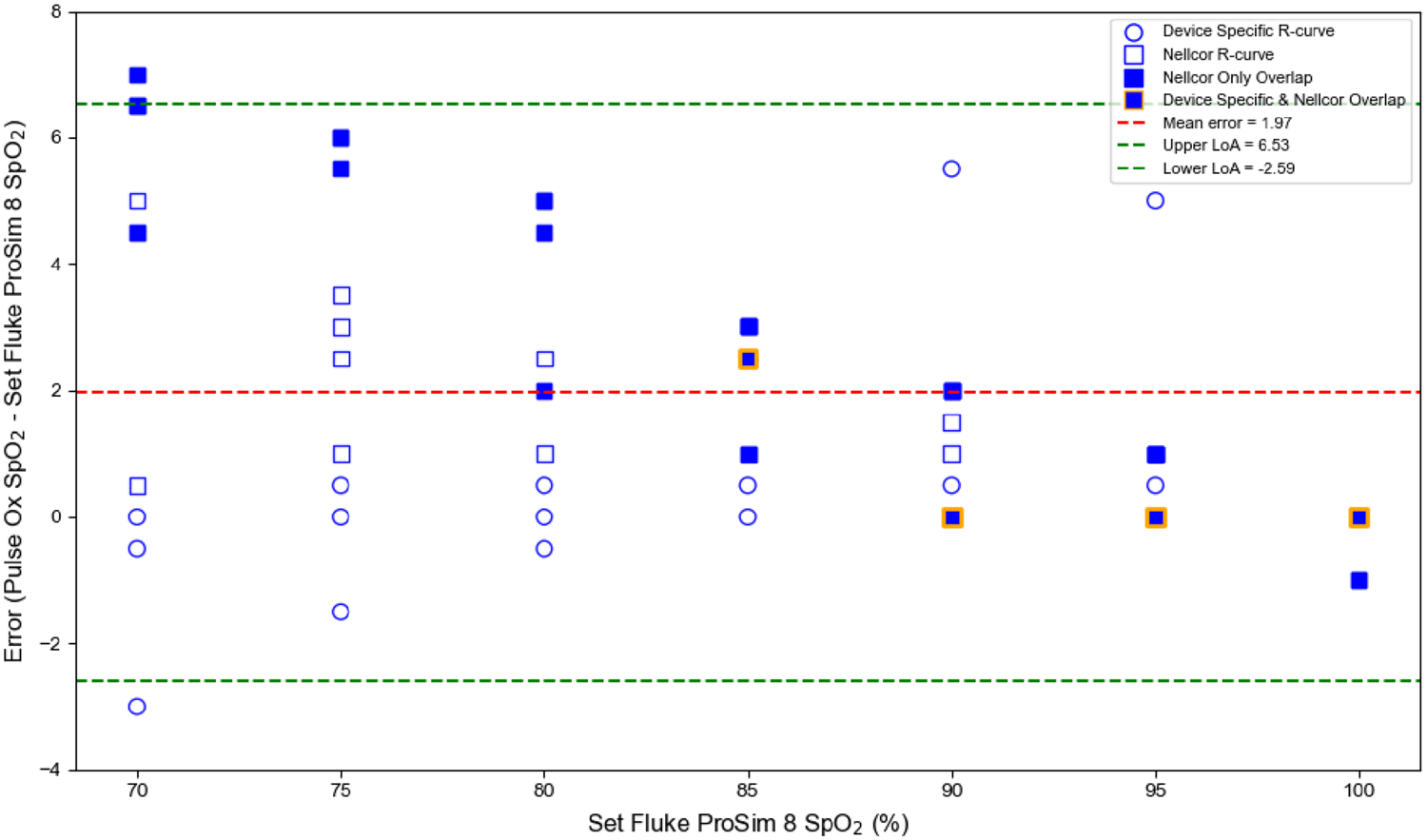
Bland-Altman plot illustrating the measurement error (bias) of 12 pulse oximeters compared to the set SpO₂ values on the Fluke ProSim 8 (FPS8). For three devices – the Nonin CO-Pilot, Masimo MightySat, and Nonin Onyx Vantage 9590 – their device-specific R-curves were used and are displayed as circles. The remaining nine devices, which did not have manufacturer specific R-curves programmed on the FPS8, were analyzed using the default Nellcor R-curve and are displayed as squares. When more than one square-marked devices had the same error value, the overlapping points are shown as a blue-filled square. When a square-marked device exhibited an error value that overlapped with that of a circle-marked device, the data point is shown as a blue-filled square with an orange outline. Error was defined as the difference between the pulse oximeter SpO₂ and the set FPS8 SpO₂, and is plotted against the set FPS8 SpO₂. The dashed red line represents the mean bias (mean error = 1.97), and the dashed green lines indicate the upper and lower limits of agreement at 6.53 and − 2.59, respectively. This plot was generated using Matplotlib

Of these six, three failed to meet performance recommendations and standards during controlled desaturation studies in healthy human adults (i.e. had an ARMS > 3%) (Table 2). At the lower set SpO₂ levels on the FPS8, the oximeters consistently reported higher SpO₂ values than the set SpO₂ on the Fluke. (Fig. 1).

For example, at a set SpO_2_ of 70% on the FPS8, eight of the 12 devices tested overestimated the set SpO_2_ on the FPS8 by more than 3% (Fig. 1). The Biolight M70 demonstrated the highest ARMS value (ARMS = 5.08) among all devices during the controlled desaturation study in humans, yet it achieved the lowest ARMS (ARMS = 0.96) during the SpO₂ plateau protocol using the FSP8 (Table 2). Similarly, the Nonin CO-Pilot had the best performance during the controlled desaturation study (ARMS = 1.39) but performed poorly using the SpO₂ plateau protocol with ARMS values of 3.49 and 3.68, when using the manufacturer-specific R-curve and the default Nellcor R-curve, respectively (Table 2).

**Fig. 2:**
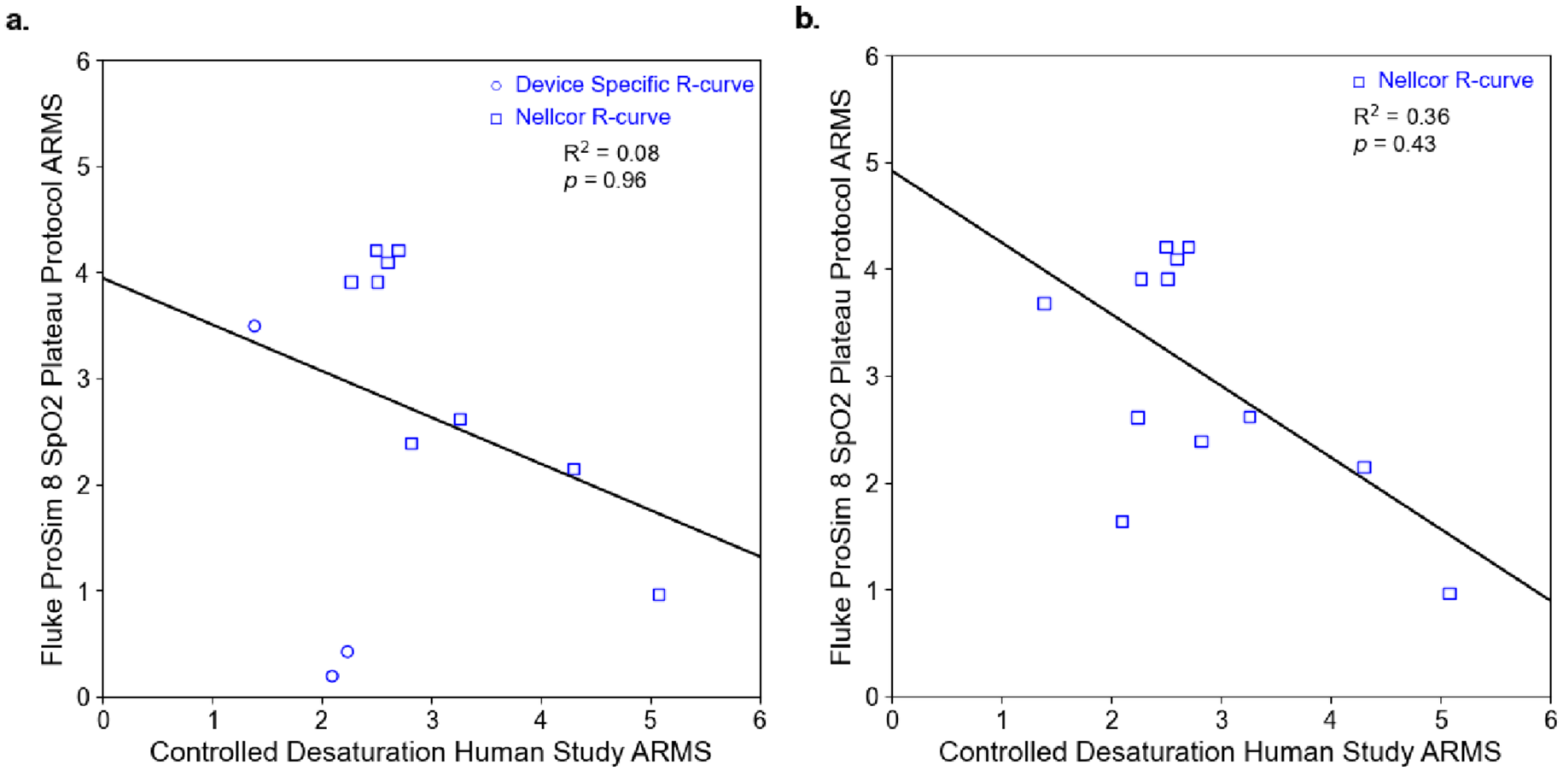
This figure compares the ARMS (accuracy root-mean-square) values of 12 pulse oximeters obtained from the Fluke ProSim 8 (FPS8) SpO₂ plateau protocol with those calculated from controlled desaturation studies in humans. Panel (**a**) includes ARMS values from three pulse oximeters—Nonin CO-Pilot, Masimo MightySat Rx, and Nonin Onyx Vantage 9590—for which device-specific R-curves were available and used during FPS8 testing (shown as open circles), along with nine other devices tested using the default Nellcor R-curve (shown as open squares). Panel (**b**) presents ARMS values for the same 12 devices, all tested using the default Nellcor R-curve. In both panels, the solid black line represents the linear regression between FPS8 ARMS and human controlled desaturation studies ARMS. The coefficient of determination (R²) and p-value are displayed in the upper right corner of each plot. This plot was generated using Matplotlib

There was no statistically significant relationship between device performance during the controlled desaturation studies and their performance on FPS8 during the in vitro SpO_2_ plateau protocol, either with the manufacturer-specific R-curve (*p* = 0.96) or with the default Nellcor R-curve (*p* = 0.43) (Fig. 2).

When testing the Nonin CO-Pilot, Masimo Mightysat, and Nonin Onyx Vantage 9590 using their manufacturer-specific R-curve versus the default Nellcor R-curve, we found that the Masimo Mightysat and Nonin Onyx Vantage 9590 had large improvements in their performance on the FPS8 when their R-curve was used, while the Nonin CO-Pilot performed similarly with its manufacturer-specific R-curve and the default Nellcor R-curve on the FPS8 (Table 2).

## Signal space protocol

**Fig. 3:**
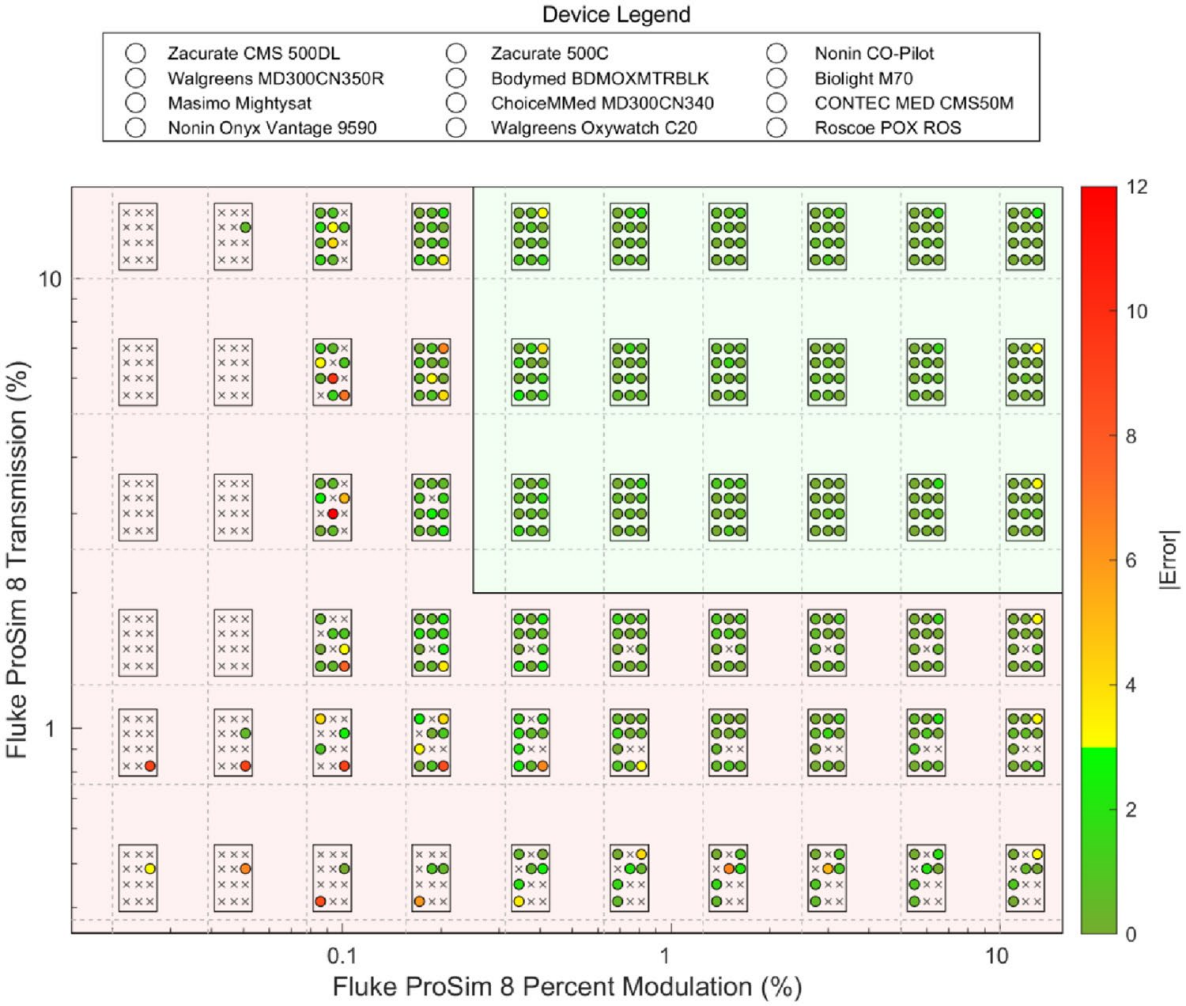
Range of transmission and modulation values tested during the signal space plateau protocol for each of the 12 pulse oximeters. The position of each pulse oximeter on the device legend corresponds to the same positioning within each box (e.g. the top left circle or ’x’ within every box corresponds to the Zacurate CMS 500DL). The light green background shading indicates high signal strength (transmission values *≥* 2.5% and percent modulation values *≥* 0.313), while the light red background shading represents the low signal space (transmission < 2.5% and percent modulation values < 0.313). The intersections of the dashed lines represent the specific FPS8 transmission and modulation combinations tested. The circle scatter points are color coded based on the absolute value of the error (difference between the reported SpO_2_ and the setpoint of the Fluke) at each test point. Scatter points that are colored green represent devices that performed well (+/- 0–2.99.99% error). The orange represents devices that show moderate degrees of error (+/- 3–7% error). The orange to red coloring represents device performance with large errors (> 7% error and unstable drifting values). Scatter points with an ‘x’ represent test points where the devices failed and did not report an SpO_2_. MATLAB software was used to create this figure

On the signal space protocol, all the devices tested on the FPS8 performed well (+/- 0–2.99.99% error) under high signal space (Fig. 3).

However, a majority of devices tested in the low signal space either failed to provide a reading or displayed significant errors in SpO_2_ (i.e. error > 3%) (Fig. 3). Across the 60 modulation and transmission pairings per device, the pulse oximeters failed to produce a reading an average of 20.2 ± 7.2 times, accounting for over one-third of the total testing parameters. (Table 2). As the modulation and transmission values decreased, the frequency of pulse oximeter reading failures increased (Fig. 3). In general, the ARMS was higher for the low signal space compared to the high signal space.

**Fig. 4:**
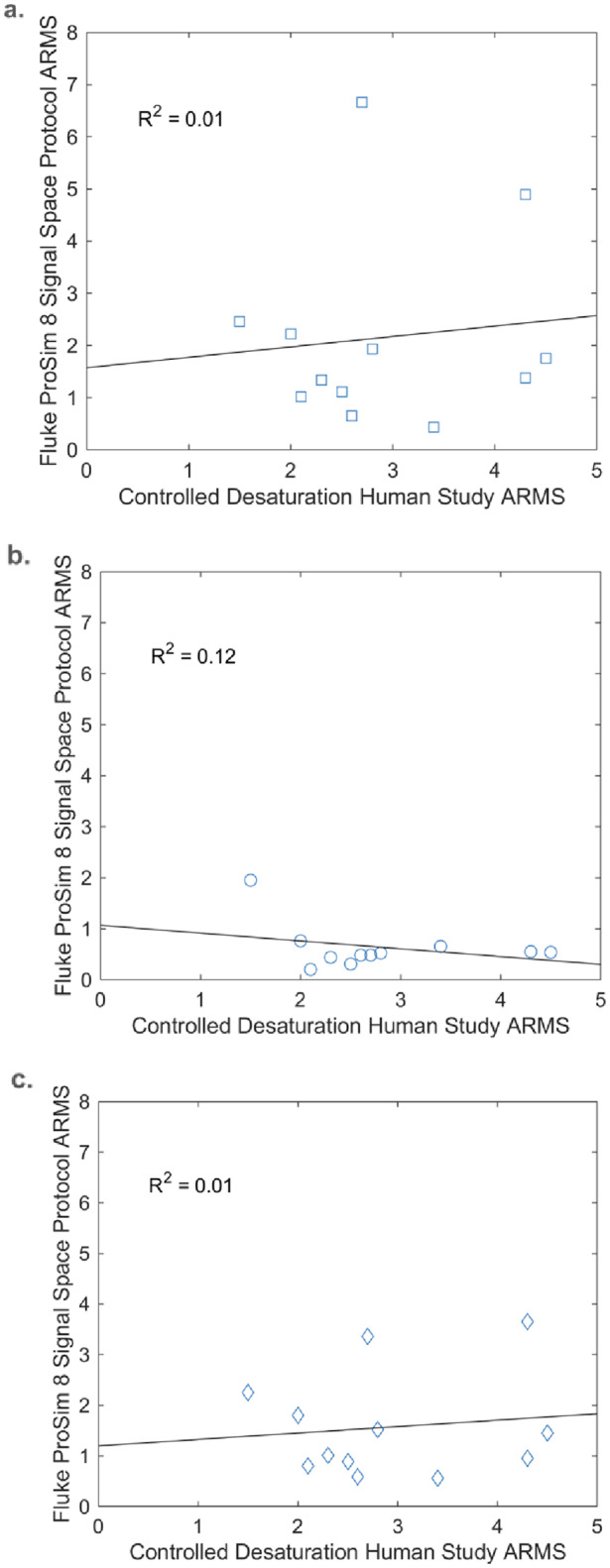
Calculated ARMS of each pulse oximeter from the FPS8 signal space protocol testing plotted with respect to the calculated ARMS from a hypoxia study. The ARMS was calculated for the FPS8 testing at three different conditions: (**a**) low signal strength, (**b**) high signal strength, and (**c**) all the test points. The black dashed line represents the linear regression between the FPS8 ARMS and hypoxia study ARMS with the coefficient of determination (R^2^) for the regression shown in the upper left of each plot. MATLAB software was used to create this figure

Across both signal spaces, the oximeter performance during FPS8 testing did not correlate to the controlled desaturation study testing in healthy humans as seen by the overall R^2^ = 0.01 (Fig. 4).

**Fig. 5:**
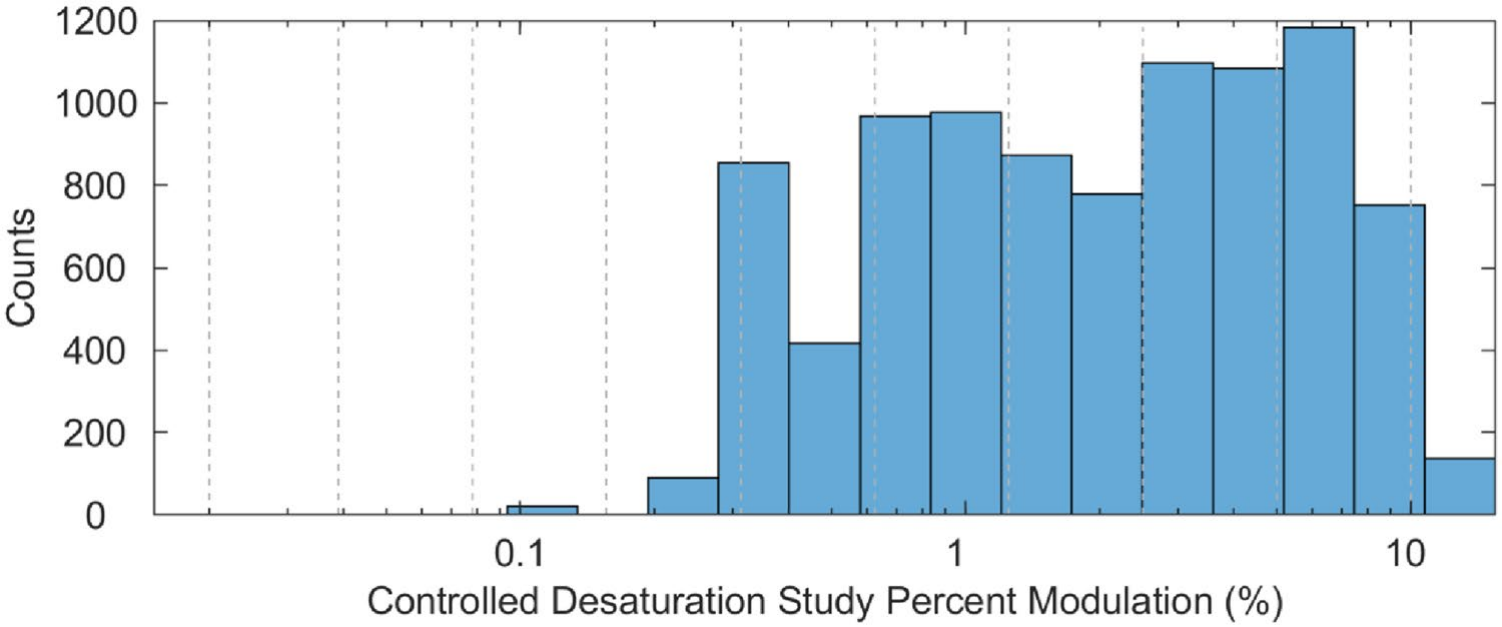
This histogram displays the frequency (counts) of the recorded percent modulation values from the twelve pulse oximeters during controlled desaturation studies. The grey dashed lines indicate the specific percent modulation levels tested using the FPS8 device: 10.0, 5.0, 2.5, 1.25, 0.625, 0.313, 0.156, 0.078, 0.039, and 0.020. MATLAB software was used to create this figure

The best-performing devices in the human controlled desaturation studies did not align with the best-performing devices on the functional tester when using the signal space protocol (Table 2). Similarly, poor performance on the functional tester did not match poor performance in human testing (Table 2). In addition, the percent modulation values recorded by the pulse oximeters during the controlled desaturation study were generally comparable to those applied during the signal space protocol; although the lowest percent modulation levels tested (0.078%, 0.039%, and 0.020%) were not observed during the human testing (Fig. 5).

During the desaturation studies, the percent modulation ranged from 0.1% to 14.1% and the mean percent modulation across all devices tested were between 2.13% and 3.57%.

Of the 12 devices tested using the signal space protocol, 10 passed testing on the FPS8 (i.e. ARMS *≤* 3%). However, two of these 10 devices failed the controlled desaturation studies (i.e. had ARMS ≤ 3%). Based on the Open Oximetry database at the time of publication, nine of the oximeters we tested passed 2017 ISO (ARMS ≤ 4%) and 2013 FDA (ARMS ≤ 3%) regulatory frameworks, while three did not [38] (Table 2). Of the three that failed the human studies, the signal space protocol only correctly identified one of the three devices.

## Discussion and conclusion

We used two novel protocols with a functional tester to assess pulse oximeter performance and found neither protocol was able to predict oximeter performance in humans during controlled hypoxemia. We also found that the FPS8 was unable to reliably identify differences in oximeter performance under low signal conditions, as many devices either failed to display a SpO₂ reading or displayed significant errors. These findings support the notion (and warnings by manufacturers) that functional testers should not be used to validate the accuracy of SpO_2_ which could lead to false reassurances of device performance in clinical settings and wrongly justify procurement of low-quality, inaccurate devices, especially in low-income countries.

Few prior studies exist for comparison. One such study compared pulse oximeter performance on the Lightman functional tester against measured SaO₂ in human adult patients (18–80 years old) requiring intensive or high dependency care in a prospective observational study [28]. This study tested the LED wavelength accuracy of three reusable pulse oximeter probes on the Lightman functional tester, categorizing them as accurate, under-reading, or over-reading based on their degree of errors at SpO₂ of 97%, 90%, 80%, and 70% on the Lightman [28]. The study demonstrated that the Lightman functional tester can identify faulty probes; however, this study was limited to three reusable oximeter probes, all from the same manufacturer [28]. Multiple additional studies have employed functional testers to evaluate pulse oximeter SpO₂ performance, measuring how well the pulse oximeter under test was able to report the SpO₂ on the functional tester, without directly validating these readings against measured SaO₂ levels [6–11, 22, 25, 26]. Our results confirm that functional testers should not be used to verify pulse oximeter SpO₂ accuracy, and we are unaware of any existing data that contradicts this conclusion.

**Fig. 6:**
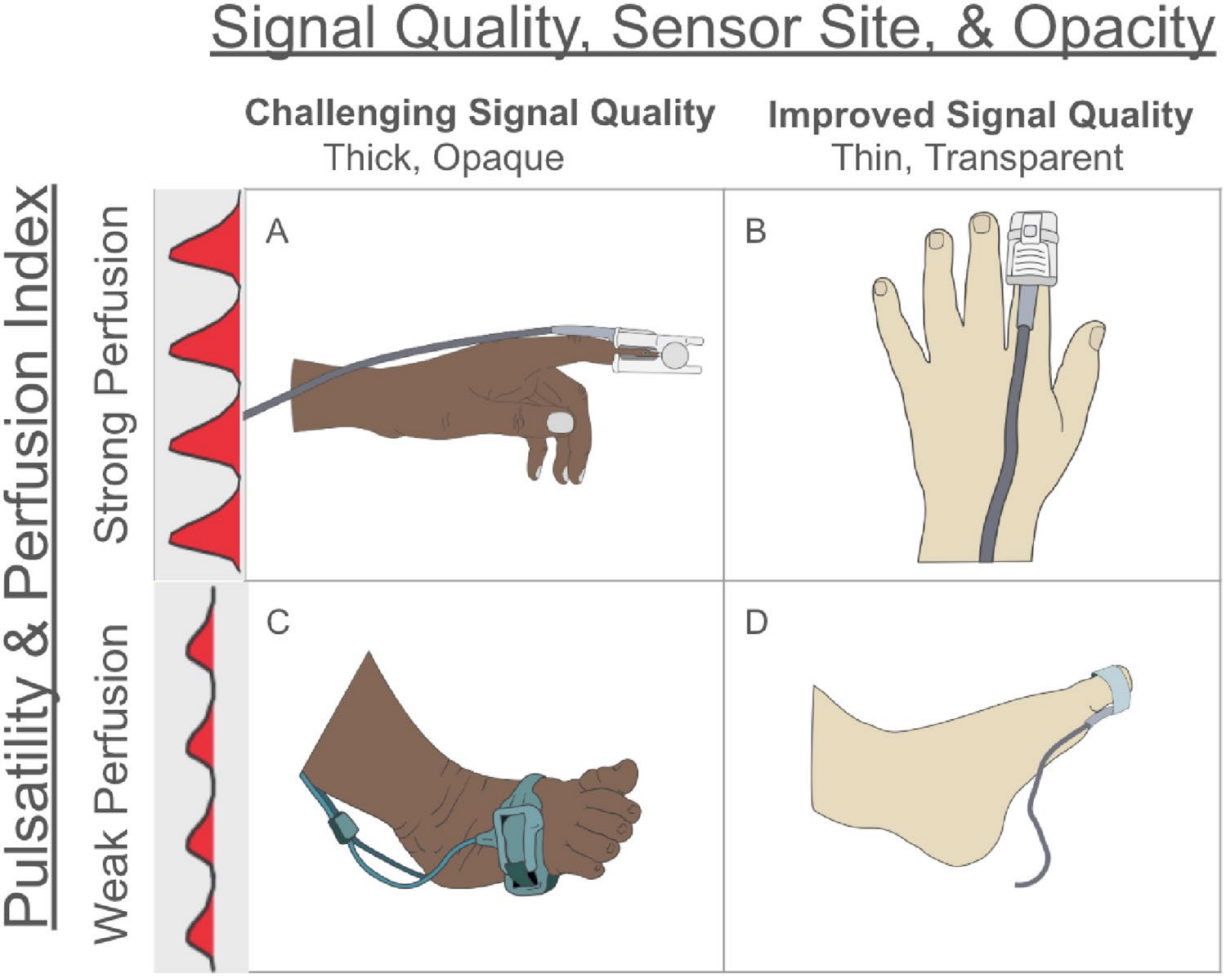
Pulse oximeter signal quality depends upon numerous factors; two critical factors include the sensor site and skin color. Cell B demonstrates optimal signal quality for pulse oximeters since the device is used on a thin finger with a light skin color. Cells A and D represent an intermediate level of pulse oximeter signal quality; cells A and D are challenged at similar levels but in different ways. In cell A, the probe is located on a finger with strong perfusion but is impacted by the skin tone and finger thickness. Cell D represents another intermediate accuracy level where the skin site is lightly pigmented, but the perfusion is poor. Cell C signifies the least favorable conditions for pulse oximeter signal quality because the probes are utilized on an area that is thick, poorly perfused, and darkly pigmented. Google slides was used to create this figure, and the images were sourced from the Open Oximetry database [38]

There are several potential explanations for our findings that performance during in vitro testing did not correlate with performance during desaturation studies. First, controlled desaturation studies include only healthy adults for safety and ethical reasons. It is highly likely that these healthy subjects exhibit signals greater than the high signal space testing points of the FPS8 (i.e. values of transmission > 1% and percent modulation > 0.5). This, coupled with the fact that the FPS8 cannot replicate the probe-tissue optical interactions in humans contributed to the lack of correlation between the FPS8 testing and human testing. When pulse oximeters are evaluated on healthy subjects during human studies, the signals that the oximeters process are constrained to only a subset of the signals present during the in vitro test. In the clinical setting, however, many factors (e.g., the sensor’s location, pulsatility, skin color, finger thickness, and disease states) impact the signal quality of pulse oximeters (Fig. 6).

Another potential explanation for why functional tester findings may differ from results in human studies is that the functional tester utilizes its own LEDs and does not rely on the LEDs of the oximeter under test. In other words, if the oximeter has faulty LEDs, their potential negative impact of performance may not be seen.

This study had several limitations. We only used one functional tester and 12 oximeters, which may have limited our ability to detect significant correlations, especially if device performance differences were subtle. In addition, these results may not be generalizable to other functional testers or oximeters on the market (Table 1). Although not all functional testers are oximeter-specific, the FPS8 had manufacturer-specific R curves for only three of the 12 devices tested. Thus, for the nine devices without an R-curve, the data we collected may not accurately reflect their performance. Additionally, even though the signal space protocol was designed by a group of expert pulse oximeter engineers to evaluate pulse oximeter performance, the thresholds and settings chosen may not have been ideal for detecting the intended effects, given that the three lowest percent modulation values tested during the signal space protocol were not recorded during the controlled desaturation studies (Fig. 5). As such, we acknowledge that the comparability between the simulator-based and human desaturation data is limited, particularly because the controlled desaturation studies did not achieve perfusion or modulation levels as low as those simulated by the FPS8. This discrepancy may have reduced the direct translatability of the simulator results to physiologic conditions encountered in human testing.

While our protocols show that the functional tester cannot reliably validate SpO₂ accuracy against SaO₂ values, a validated functional tester protocol would be highly valuable. Further research into alternative functional tester designs and protocols could improve identification of poorly performing devices, streamlining research and reducing time and costs. Until a validated protocol exists, functional testers should not be used to verify the accuracy of pulse oximeters.

## Data Availability

The research data supporting the results of this manuscript are available upon request. Interested researchers can contact the corresponding author at seif.elmankabadi@ucsf.edu for access to the data. All data supporting the findings of this study are openly accessible through the Open Oximetry Data Repository. Data from controlled desaturation studies are de-identified and shared under the repository’s terms, ensuring adherence to data collection protocols, local IRB approvals, and other relevant regulations. Users can access the data by creating a PhysioNet account and agreeing to the repository’s terms of use. For access details, please visit the [Open Oximetry website](https:/openoximetry.org/data-repository).
